# Isolation and Identification of *Bacillus aryabhattai* M2C: Its Effects With Vermicompost on Yield and Nutrients of Peanut (*Arachis hypogaea* L.)

**DOI:** 10.1155/ijm/9923279

**Published:** 2025-11-10

**Authors:** Nguyen Van Chuong, Tran Le Kim Tri, Tran Minh Vu, Le Minh Tuan, Tran Thanh Liem, Nguyen Ngoc Phuong Trang

**Affiliations:** ^1^An Giang University, An Giang, Vietnam; ^2^Vietnam National University, Ho Chi Minh City, Vietnam

**Keywords:** biofertilizer, endophytic bacteria, peanut, sustainable agriculture, vermicompost

## Abstract

A native strain of *Bacillus aryabhattai* M2C was isolated from peanut root nodules and confirmed for its ability to fix atmospheric nitrogen under controlled laboratory conditions. To assess its agronomic potential, field experiments were conducted to evaluate the combined effects of bacterial inoculation and vermicompost on peanut growth, yield, and seed nutritional quality. The trial was arranged in a randomized complete block design with two factors: (i) bacterial inoculation (with or without *B. aryabhattai* M2C) and (ii) three vermicompost levels (0, 5.0, and 10.0 t ha^−1^), resulting in six treatment combinations with four replications each. Growth traits, fresh pod yield, and seed nutrient composition were systematically measured. Results showed that although interactions between bacterial inoculation and vermicompost application were not significant for fresh pod yield, inoculation with *B. aryabhattai* M2C alone increased yield by 7.43% compared with the uninoculated control. Vermicompost at 5.0 and 10.0 t ha^−1^ did not differ significantly from each other, but both outperformed the untreated control by 6.12% and 8.81%, respectively, while also improving vegetative growth, biomass accumulation, and seed nutritional quality. Despite the inconsistent interactions across all traits between strain M2C and vermicompost, the bacterial strain combined with vermicompost application contributed to yield improvements compared with the control treatments. The integration of strain M2C with 5.0 t ha^−1^ vermicompost appears to be the optimal strategy, reducing vermicompost input while sustaining yield gains. Overall, this integrated approach represents a sustainable and eco-friendly alternative to chemical inputs, with strong potential to improve peanut productivity and support long-term soil fertility management in agricultural ecosystems.

## 1. Introduction

Groundnut (*Arachis hypogaea* L.) is a globally significant leguminous crop, prized for its adaptability to a wide range of soil types and climatic conditions. As a primary source of edible oil and protein, peanuts contribute substantially to both human nutrition and livestock feed, especially in tropical and subtropical regions where dietary protein and healthy fats are often limited. In Vietnam's Mekong Delta, including An Phu district, groundnut cultivation is integral to smallholder farming systems, offering both economic returns and nutritional benefits that help improve rural livelihoods [1–3]. Despite its value, groundnut production faces persistent agronomic and environmental challenges. Farmers contend with issues such as climate variability, declining soil fertility due to overuse of chemical fertilizers, and the prevalence of soil-borne diseases [4, 5]. The decline in soil microbial diversity is particularly detrimental, as microorganisms play critical roles in nutrient cycling, organic matter decomposition, and the suppression of soil-borne diseases [6, 7]. Loss of functional microbial groups can disrupt ecological balance, reduce nutrient availability, and compromise soil structure, ultimately limiting plant growth and yield potential [8, 9]. Moreover, the depletion of beneficial microbes increases plant vulnerability to pathogenic infections, further constraining productivity in intensive agroecosystems. Restoring soil microbial diversity is therefore essential for maintaining soil health, enhancing crop resilience, and achieving sustainable agricultural production [10, 11].

Soil nitrogen (N) deficiency remains a major constraint limiting peanut productivity. Previous studies have shown that insufficient N availability significantly reduces yield and seed quality [12, 13]. The decline in soil fertility, together with the intensive use of chemical fertilizers, highlights the urgent need for sustainable management strategies that enhance biological N fixation, improve nutrient cycling, and reduce dependence on synthetic inputs [14, 15]. Numerous studies have emphasized the benefits of combining organic fertilizers with microbial inoculants to improve soil fertility, microbial diversity, and crop yield [16–18]. However, smallholder farmers often face challenges in accessing high-quality microbial products and balancing trade-offs between chemical, organic, and biological inputs [19, 20]. The integration of legume crops with N-fixing bacteria provides a sustainable solution by supplying organic N to the soil, enhancing soil structure, and restoring degraded ecosystems [21, 22]. Recent research demonstrates that inoculation with N-fixing bacteria significantly increases nodule formation, N fixation, and overall plant vigor compared to noninoculated controls. These bacteria not only supply organic N to the crop but also contribute to improved soil structure and resilience against pathogens [18, 20, 23]. Among organic amendments, vermicomposting has gained attention for its ability to improve soil aeration, nutrient content, and water retention. Rich in humus and microbial biomass, vermicompost enhances nutrient uptake and promotes root development, thereby contributing to higher productivity in leguminous crops [24, 25]. The synergistic effect of combining vermicompost with other organic or biological inputs has been shown to enhance soil properties such as pH, electrical conductivity, and the availability of key nutrients, thereby creating a more favorable environment for groundnut growth. Biological control agents, such as *Bacillus aryabhattai*, have also been successfully deployed to manage root-rot diseases in peanuts [26, 27]. Field trials in India demonstrated that the application of beneficial endophytic *Bacillus* strains effectively suppressed root rot incidence while enhancing crop yields compared with conventional farmer practices. The participatory introduction of such biocontrol agents has promoted their acceptance among smallholder farmers, resulting in improved plant health, reduced reliance on chemical inputs, and increased farm profitability [28, 29]. Despite the clear benefits of integrating organic and biological inputs, smallholder farmers often face barriers to adoption. These include limited access to high-quality microbial inoculants, knowledge gaps regarding optimal application methods, and the challenge of balancing chemical, organic, and biological inputs within their production systems [30, 31]. Integrating leguminous crops with N-fixing bacteria and organic amendments provides an ecologically sustainable strategy to restore soil functionality, improve crop productivity, and mitigate adverse environmental impacts [32].

Recent studies emphasize the importance of holistic soil management strategies that combine organic fertilizers, microbial inoculants, and reduced chemical inputs. Such approaches not only improve soil fertility and plant nutrition but also foster greater resilience against pests and diseases, ultimately supporting more sustainable and profitable groundnut production systems [27, 33]. Vermicompost is rich in essential plant nutrients such as N, phosphorus (P), potassium (K), calcium (Ca), manganese (Mn), zinc (Zn), and copper (Cu), along with other elements that enhance plant nutrition, photosynthesis, leaf chlorophyll levels, and the nutrient composition of various plant parts [34, 35]. Inoculating peanut genotypes with specific bacterial strains resulted in increased nodule count, nodule biomass, and N fixation compared to uninoculated controls, demonstrating the effectiveness of these bacteria over conventional chemical fertilizers. Additionally, bacterial inoculants are more cost-effective and environmentally friendly, as they do not cause contamination after application, unlike chemical fertilizers [36, 37]. By exploring these integrated approaches, the study is aimed at (i) advancing sustainable peanut cultivation, offering both environmental and economic benefits for smallholder farmers facing the complex challenges of modern agriculture and (ii) seeking to build on these advances by investigating the combined effects of vermicompost application and inoculation with N-fixing bacterium strain M2C on the yield, productivity, and nutrient content of peanuts grown under low-fertility conditions. By addressing the dual challenges of declining soil health and chemical input dependence, the research is aimed at providing practical solutions for resource-limited farmers and contributing to the broader goal of sustainable agriculture in the Mekong Delta and similar agroecological zones.

## 2. Materials and Methods

### 2.1. Isolation and Molecular Identification of *B. aryabhattai* M2C

Nodules of peanut plants were collected and subjected to surface sterilization using 96% ethanol for 15 s, followed by immersion in 4% sodium hypochlorite for 5 min. The sterilized tissues were rinsed multiple times with sterile distilled water to ensure complete removal of residual disinfectants, as described by Chandran et al. [37]. Subsequently, nodules were macerated in a drop of 0.9% sodium chloride (NaCl) using sterile equipment, and the suspension (0.1 mL) was spread onto yeast mannitol agar (YMA) plates. Cultures were incubated at room temperature for 4 days to allow the growth of bacterial colonies. Single colonies were selected and further characterized through Gram staining and standard biochemical tests, including catalase and oxidase activity, salt tolerance, and motility assessment [38, 39]. The isolates that exhibited traits consistent with N-fixing endophytic bacteria were selected for molecular identification. Genomic DNA was extracted, and the 16S rRNA gene was amplified and sequenced. Sequence alignment was performed using NCBI BLAST, and strain M2C showed 100% similarity with known N-fixing bacteria in the database [40].

### 2.2. 16S rRNA Gene Sequencing

Genomic DNA was extracted from pure colonies placed in Eppendorf tubes using the GeneJET Genomic DNA Purification Kit (Thermo Scientific). The 16S rRNA gene was amplified by PCR with the primer pair 20F (5⁣′-CTACGGCAAGGCGACGCTGACG-3⁣′) and 1500R (5⁣′-GGTTACCTTGTTACGACTT-3⁣′) [40]. Sequence analysis was performed using MEGA software, and BLAST comparison against the GenBank database revealed a 99.86% similarity with the closest sequence, deposited under Accession Number KU161278.1 [41].

### 2.3. Thermal Tolerance Assessment

The thermal adaptability of strain cjy13 was examined in two stages. Initially, YMA nutrient broth was dispensed into test tubes assigned for each strain. Subsequently, cultures were streaked onto YMA agar slants and incubated at three different temperatures: 25°C, 37°C, and 45°C. Each condition included four replicates, and colony growth was evaluated over a period of 7 days [40].

### 2.4. Salt and pH Tolerance

Salt tolerance of strain cjy13 was determined by supplementing liquid YMA medium with NaCl at concentrations of 0.5%, 2%, 3%, 4%, and 5%. Each of the four strains was inoculated separately into these media and maintained at 28°C. Growth performance was monitored after 1 week, with four replicates per treatment [40]. For pH tolerance testing, the liquid YMA medium was adjusted to pH 4.5 using 0.1 N HCl and to pH 7.0 and 8.5 using 0.1 N NaOH, followed by verification with a calibrated pH meter. Cultures were inoculated and incubated at 28°C. Each treatment included four replications, and bacterial development was assessed after 7 days [40].

### 2.5. Ammonia Production

The qualitative ability of the strain M2C to produce ammonia was assessed by inoculating cultures into peptone water and incubating on YMA medium at 30°C for 60–80 h. The formation of ammonia was indicated by a distinct color change from brown to yellow following the addition of Nessler's reagent [12, 42].

### 2.6. Nitrogenase Activity

Nitrogenase activity of the isolate was determined using the acetylene reduction assay (ARA) as described by Soper et al. [43]. *B. aryabhattai* M2C was precultivated in YMA broth for 24 h, adjusted to OD_600_ = 0.8, and transferred into N-free medium. Cultures were incubated at 30°C with shaking at 160 rpm, after which acetylene was introduced into the vial headspace. Following incubation, gas samples were analyzed by gas chromatography, and ethylene concentrations were quantified using an external calibration curve. Results were expressed as nmol C_2_H_4_ h^−1^ mL^−1^. Negative (noninoculated) and positive controls were included, each with four independent replicates to ensure reproducibility.

### 2.7. N Concentration Analysis

To quantify N, *B. aryabhattai* M2C was grown in N-free medium supplemented with 0.05% malate as the sole carbon source and incubated at 30°C. Cultures were centrifuged at 3000 rpm for 1 min, and the resulting supernatants were collected and analyzed following the protocol of Daoliang et al. [44].

### 2.8. Preparation and Inoculation of *B. aryabhattai* M2C

The strain of *B. aryabhattai* M2C was cultured in 250-mL Erlenmeyer flasks containing 200 mL of diluted YMA broth. Cultures were incubated on a rotary shaker at 100 rpm and 20°C for 12 h. Cells were harvested by centrifugation at 13,000 rpm for 1 min at 2°C and washed twice with sterile distilled water. The final suspension was adjusted to a concentration of approximately 10^8^ CFU mL^−1^.

Groundnut seeds (cv. L18, obtained from the Vietnam Seed Company) were surface-sterilized and soaked in the bacterial suspension for 5 min. Treated seeds were left to air-dry in the dark for 12 h before sowing. Additional bacterial inoculations were applied at 20, 40, and 60 DAS in the treatments with bacterial strains to maintain a stable bacterial population in the soil [45].

### 2.9. Field Experimental Design

A field experiment was conducted from January to April 2025 in An Phu commune, Vietnam. The study was arranged in a two-factor factorial design with four replications, consisting of (i) bacterial inoculation (with and without *B. aryabhattai* M2C) and (ii) vermicompost (VA) at three levels (0, 5.0, and 10.0 t ha^−1^). In total, six treatments were established (Table 1), each replicated four times, giving a total experimental area of 480 m^2^ (2 × 10 m × 4 replications × 6 treatments). Groundnut seeds were sown at a spacing of 25 × 30 cm, with two seeds per hole, and thinned to one healthy plant 15 days after sowing. All treatments received chemical fertilizers at rates of 40 kg urea N ha^−1^, 60 kg P_2_O_5_ ha^−1^, and 60 kg KCl ha^−1^, which were supplied by the Binh Dien Fertilizer Company, Vietnam.

The vermicompost supplied by SFARM Company (Vietnam) contained 1.08% total N, 0.95% P, 0.39% K, 1.28% Ca, and 0.47% Mg. The initial soil characteristics in An Phu were as follows: pH 5.07, cation exchange capacity (CEC) 2.03 cmol^+^ kg^−1^, soil organic matter (SOM) 3.24%, total N 0.125%, available P 259 mg kg^−1^, and exchangeable K 85.1 mg kg^−1^. The soil texture was classified as silt sandy loam, consisting of 25.1% silt, 68.5% sand, and 6.4% clay, and was considered suitable for peanut cultivation.

### 2.10. Data Collection and Analysis

Agronomic traits such as biomass, pod number, pod weight, nodulation (number and weight of nodules), and 100-seed weight were recorded at harvest. Fresh pod yield (t ha^−1^) was calculated for each treatment. In addition, biochemical analyses of seeds were conducted to determine lipids, protein, N, P, and K content using standard protocols. All data were analyzed using Statgraphics XV and Microsoft Excel. Analysis of variance (ANOVA) was conducted, and treatment means were compared using the least significant difference (LSD) test at a significance level of *p* ≤ 0.05.

## 3. Results

### 3.1. Biochemical and Molecular Characterization of *B. aryabhattai* M2C

**Figure 1:**
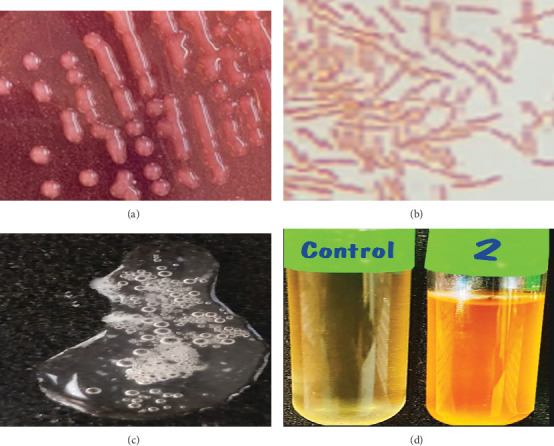
(a) Pure colony on YMA medium. (b) Gram-positive, short rod-shaped bacteria. (c) Bubble creation in reaction with catalase. (d) Ammonia produces an orange color with peptone of *B. aryabhattai* M2C (2) and control.

Figure 1a illustrates that the colonies were pure, pink, round, and slightly convex with intact margins, confirming successful isolation. Gram staining (Figure 1b) revealed short rod-shaped, Gram-positive cells, characteristic of the genus *Bacillus*. A strong catalase reaction was observed upon exposure to H_2_O_2_ (Figure 1c), indicating the ability to decompose hydrogen peroxide, a trait common among microaerophilic bacteria. In peptone medium, the strain produced an orange coloration upon reaction with ammonia (Figure 1d), demonstrating that *B. aryabhattai* can degrade organic N compounds to release ammonia, an essential component of the N cycle. Collectively, these biochemical traits support the taxonomic identification of strain M2C and highlight its potential role in agricultural applications through organic N mineralization.

**Figure 2:**
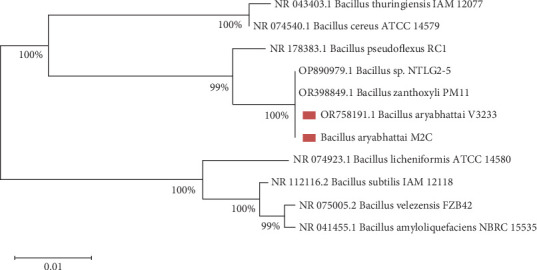
Neighbor-joining phylogenetic tree based on partial 16S rRNA sequences of *B. aryabhattai* M2C strains isolated from peanut nodules, alongside selected reference strains. Tree construction was performed using MEGA 11. Scale bar indicates 0.01 nucleotide substitutions per site, and bootstra*p* values are shown at branch nodes. GenBank accession numbers are included.

Figure 2 contains the 16S rRNA gene DNA sequence of *B. aryabhattai* M2C used for molecular identification and phylogenetic analysis. Figure 2 presents a neighbor-joining phylogenetic tree, which was constructed based on partial 16S rRNA gene sequences of *B. aryabhattai* M2C strains isolated from peanut nodules, along with several selected reference strains for comparison. The phylogenetic analysis was carried out using the MEGA 11 software, which allowed for accurate alignment and tree building. The scale bar on the tree represents 0.01 nucleotide substitutions per site, providing a measure of the genetic distance between the strains. Bootstra*p* values, which indicate the reliability of the branching points, are displayed at the nodes of the tree to support the inferred relationships. Additionally, GenBank accession numbers for each sequence are included to facilitate reference and verification. The phylogenetic tree helps to clarify the evolutionary relationships of the isolated *B. aryabhattai* M2C within the broader context of related bacterial species, supporting their taxonomic identification and potential functional characterization.

**Figure 3:**
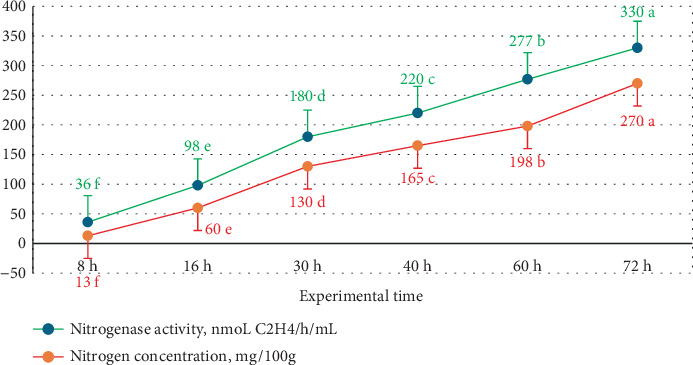
Nitrogenase activity of *B. aryabhattai* M2C evaluated via the acetylene reduction assay together with nitrogen concentration measurements. Different letters (a, b, c, d, e, and f) indicate statistically significant differences among treatments at the *p* < 0.01.

The results presented in Figure 3 demonstrated the nitrogenase activity of *B. aryabhattai* M2C, which was evaluated using the ARA. Time period analysis from 8 to 72 h showed a significant increase in nitrogenase activity, rising from 36 to 330 nmol C_2_H_4_/h/mL. This indicates a strong biological N fixation capability of this *Bacillus* strain. This activity was further supported by measurements of N concentration over the same period, which increased from 13 to 270 mg/100 g, confirming the strain's ability to convert atmospheric N into a biologically available form. The consistency between the ARA results and N quantification highlights the robust N-fixing potential of *B. aryabhattai* M2C. These findings emphasize the strain's promise as a plant growth–promoting rhizobacterium (PGPR), capable of enhancing plant growth through efficient N fixation, making it a valuable candidate for sustainable agricultural applications.

Table 2 shows the physical and chemical reactions of *B. aryabhattai* M2C. The VITEK 2 analyzer and traditional analysis showed a range of biochemical tests, evaluating the strain's metabolic capabilities and tolerances. The results indicated that *B. aryabhattai* M2C can utilize beta-xyloidine and demonstrate starch hydrolysis, as both tests yielded positive results. This strain also exhibited positive reactions for catalase, citrate use, *β*-glucosidase, mannitol, raffinose, and glucose oxidation. However, *B. aryabhattai* M2C did not utilize D-mannose, D-glucose, D-galactose, or D-ribose. Furthermore, *B. aryabhattai* M2C can tolerate NaCl concentrations between 1% and 5%, temperatures ranging from 20°C to 50°C, and pH levels from 4.0 to 8.0. The oxidase test was negative. In summary, the table provided a clear overview of the biochemical characteristics of *B. aryabhattai* M2C, highlighting its metabolic strengths and limitations.

### 3.2. Impact of *B. aryabhattai* M2C and VA on Groundnut Agronomic Traits

The results in Table 3 indicate that both *B. aryabhattai* M2C and VA had significant effects on plant height, especially at 65 DAS, while their impact on branch number and chlorophyll index was less pronounced. At 65 DAS, plant height increased significantly with increasing VA rates, where the highest value (53.5 cm) was observed at 10 t ha^−1^. The inoculation with *B. aryabhattai* M2C also led to a significant increase in plant height at all points (20, 45, and 65 DAS), with inoculated plants reaching 53.5 cm at 65 DAS compared to 50.5 cm in noninoculated controls. Although the number of branches and chlorophyll index did not show significantly significant changes in most treatments, the interaction between VA and bacterial inoculation was significant for plant height across all sampling stages.

### 3.3. Impact of *B. aryabhattai* M2C and VA on Groundnut Yield Traits and Productivity

The findings presented in Table 4 indicated that two factors, the application rate of VA and the inoculation with *B. aryabhattai* M2C, exerted significant effects on the growth and yield parameters of peanuts at the 1% significance level. Specifically, the incorporation of VA at rates of 0.0, 5.0, and 10.0 t ha^−1^ resulted in marked increases in plant biomass, the number and weight of filled pods, and the 1000-seed weight relative to the control treatment without VA. Similarly, inoculation with *B. aryabhattai* M2C positively influenced growth metrics, significantly elevating the number and weight of filled pods as well as seed weight compared to uninoculated controls. Although the interaction between VA and *B. aryabhattai* M2C was statistically significant only for the number of filled pods, the observed trends suggest a synergistic effect when both treatments are combined, leading to improved growth characteristics and yield in peanuts. These results advocate for integrated biological and organic fertilization strategies as viable approaches to sustainably enhance peanut production efficiency while minimizing environmental impact. The results presented in Table 4 demonstrated that the application of *B. aryabhattai* M2C in combination with VA at two rates of 5.0 and 10.0 t ha^−1^ did not produce significant differences in fresh yield.

### 3.4. Impact of *B. aryabhattai* M2C and VA on Groundnut Nutrient Compositions

Table 5 clearly demonstrates that both VA and inoculation with *B. aryabhattai* M2C significantly influence the nutrient composition of groundnut seeds at harvest. Notably, increasing rates of vermicompost (from 0 to 10 t ha^−1^) result in progressive enhancements in all measured seed quality traits, including lipid, protein, and macronutrient contents (N, P, and K). These improvements are significant at the 1% level, indicating that vermicompost plays a critical role in nutrient enrichment. The increase in protein content, from 16.9% at the control to 18.2% at the highest application rate, aligns with existing literature that highlights the ability of vermicompost to enhance soil biological activity and nutrient bioavailability, thereby improving seed quality. The use of *B. aryabhattai* M2C also led to a significant increase in seed nutritional parameters across the board. Treated plants show notably higher lipid and protein concentrations as well as elevated levels of N, P, and K. The significant improvement in N content from 2.73% to 2.89% following bacterial inoculation further supports the role of microbial inoculants in facilitating N uptake. Surprisingly, while both factors had significant independent effects, the interaction between vermicompost and bacterial inoculation (A × B) was only significant for seed moisture content, suggesting that their combined influence may not always be synergistic for all traits. This statistical analysis is seen in Table 5. Still, the consistent upward trends across nutrient parameters in treatments receiving both inputs underscore the value of integrated nutrient management strategies.

## 4. Discussion

*B. aryabhattai* M2C, which is a Gram-positive, rod-shaped bacterium, was isolated from peanut nodules on YMA medium. Biochemical tests confirmed positive catalase and oxidase activities, consistent with endophytic N-fixing bacteria reported in recent literature [33]. The strain demonstrated moderate salt tolerance, growing well in media containing 1%–5% NaCl, aligning with findings that most rhizobia adapt to low to moderate salinity conditions [37–40]. Molecular identification through 16S rRNA gene sequencing revealed 100% similarity with known N-fixing bacteria in the NCBI database, confirming its taxonomic position within effective rhizobial groups [46]. The strain's optimal growth temperature ranges from 20°C to 50°C, with 37°C being ideal for symbiotic N fixation, consistent with recent studies on rhizobia thermal tolerance [38]. Recent research emphasizes that *B. aryabhattai* species not only contributes significantly to N fixation under abiotic stress but also enhances plant growth by producing phytohormones and solubilizing phosphate, highlighting its potential as a biofertilizer in sustainable agriculture [47].

The nitrogenase activity observed in *B. aryabhattai M2C*, as indicated by the ARA, suggests a high potential for biological N fixation. This aligns with recent studies that emphasize the N-fixing capability of *Bacillus* genera isolated from leguminous crops [46, 47]. The significant nitrogenase activity, coupled with elevated N concentration in the assay, reflects the strain's effectiveness in contributing to N bioavailability, which is crucial for enhancing plant growth and yield. High nitrogenase efficiency of *B. aryabhattai* may be attributed to its adaptation to rhizospheric environments and its ability to interact symbiotically with legume roots [47]. Moreover, such activity is comparable to or even exceeds that of other known endophytic diazotrophs, suggesting that strain M2C could be a strong candidate for biofertilizer development [48]. These findings support the potential application of *B. aryabhattai* M2C in sustainable agriculture, particularly in reducing dependency on synthetic N fertilizers. The isolated strain M2C formed circular, pale-pink colonies on YMA medium after 4 days of incubation, consistent with typical rhizobia morphology [25, 49]. *B. aryabhattai* M2C, a Gram-positive bacterium, possesses a thick peptidoglycan-rich cell wall that retains crystal violet stain, appearing purple under microscopic examination. Biochemical tests indicated positive catalase and oxidase activity, as well as moderate tolerance to salinity (up to 5% NaCl), demonstrating adaptability to a wide range of environmental conditions. Similar results have been reported for salt-tolerant rhizobia in leguminous crops [38, 50]. The molecular identification confirmed that the strain shared 100% similarity with N-fixing rhizobacteria based on 16S rRNA gene sequencing. These characteristics support its classification as PGPR with potential for use as a biofertilizer under stress-prone environments [35, 51].

This suggests that organic amendment not only enhances nutrient availability but also positively influences vegetative growth. These findings agree with recent studies emphasizing the role of vermicompost in improving soil structure and nutrient uptake in legumes [52, 53]. This demonstrates the plant growth–promoting potential of *B. aryabhattai*, likely due to mechanisms such as phytohormone production, phosphate solubilization, or N fixation, as also noted by Fanai et al. [54] and Wu et al. [55]. This interaction effect underscores the synergistic benefits of combining organic amendments with beneficial microbes, which has been highlighted in recent integrated nutrient management research [56, 57]. Field experiments demonstrated that inoculation with *B. aryabhattai* M2C combined with VA at 10 t ha^−1^ significantly enhanced peanut yield and quality parameters compared to controls without inoculation or vermicompost. The number of filled pods per plant increased to 176 filled pods, and filled pod weight reached 196 g per plant, representing increases of approximately 31% and 65%, respectively, over untreated controls. Conversely, the number and weight of unfilled pods decreased, indicating improved pod development and resource allocation (LSD ≤ 0.01). These results align with recent findings that vermicompost improves soil nutrient availability and microbial activity, thereby promoting legume growth and yield [58]. Inoculation with strain M2C alone increased peanut yield by 12.9% compared to noninoculated treatments, while the VA of 10 t per ha raised productivity by 38.4% relative to without VA and by 11% compared to 5 t VA per ha. This synergy suggests that biological N fixation coupled with organic amendment optimizes nutrient supply and plant growth, consistent with recent integrated nutrient management studies [59, 60].

The integration of *B. aryabhattai* M2C inoculant with VA offers a promising approach to enhance peanut yield and protein content while reducing dependence on chemical fertilizers. This strategy supports sustainable agriculture by improving soil fertility and alleviating the environmental impacts associated with excessive fertilizer use [61, 62]. Adoption of biofertilizer and organic amendment combinations is recommended for peanut cultivation on nutrient-poor soils to enhance productivity and profitability for farmers while maintaining ecological balance. This effect likely stems from the bacterium's N-fixing and P-solubilizing capabilities, which facilitate enhanced nutrient uptake by the plant, as supported by recent studies on PGPR in sustainable agriculture [63]. These findings are consistent with prior studies suggesting that PGPR could improve nutrient assimilation through mechanisms such as N fixation and P solubilization [64, 65]. These results collectively emphasize the potential of combining organic amendments with beneficial microbes to sustain the nutritional quality of legume crops: a strategy increasingly advocated in modern agroecology for improving food quality and soil health [66–68].

## 5. Conclusion

*B. aryabhattai* M2C was successfully isolated, identified, and characterized, demonstrating strong potential to enhance peanut productivity under field conditions. Inoculation with strain M2C markedly improved biomass accumulation, yield components, nutrient uptake, and seed nutritional quality, particularly in terms of lipid and protein content. Although *B. aryabhattai* M2C did not consistently interact with all VA levels, inoculation of *B. aryabhattai* increased fresh pod yield by 0.75 t ha^−1^ compared with the uninoculated control. Moreover, VA application at 5 t ha^−1^ performed comparably to 10 t ha^−1^, while further improving fresh pod yield by 0.60 and 0.89 t ha^−1^, respectively. These results highlight the synergistic advantages of combining beneficial microorganisms with organic amendments in promoting peanut growth and yield performance. Long-term and multistrain field evaluations are recommended to validate the stability and broader applicability of this integrated approach for sustainable peanut cultivation.

## Figures and Tables

**Table 1:** VA rates and method of *B. aryabhattai* M2C inoculant for each treatment.

**Treatments**	***B. aryabhattai* M2C (10** ^**8**^ **CFU mL**^**−1**^**)**	**VA (t ha ** ^**−1**^ **)**	**Chemical fertilizer (kg ha ** ^**−1**^ **)**
1	No	0	40 urea–60 P_2_O_5_–60 KCl
2	Yes	0	
3	No	5	
4	Yes	5	
5	No	10	
6	Yes	10	

**Table 2:** Physical and chemical profiles of *B. aryabhattai* M2C.

**Biochemical test**	***B. aryabhattai* M2C**	**Biochemical test**	***B. aryabhattai* M2C**
Beta-xyloidine	+	NaCl (1%–5%)	++
D-Mannose	−	Temperature (20°C–50°C)	++
D-Glucose	−	pH (4.0–8.0)	++
D-Galactose	−	Citrate use	++
D-Ribose	−	*β*-Glucosidase	+
Catalase	++	Mannitol	+
Oxidase	−	Raffinose	+
Starch hydrolysis	+	Glucose oxidation	+

**Table 3:** *B. aryabhattai* M2C and VA on agronomic components in 20, 45, and 65 DAS.

**Factors**	**Plant height (cm)**			**Branch number (branches plant ** ^**−1**^ **)**			**Chlorophyll index**		
	**Days after sowing (DAS)**								
	**20**	**45**	**65**	**20**	**45**	**65**	**20**	**45**	**65**
*B. aryabhattai* M2C (A)									
No	11.5 ± 0.33b	20.7 ± 0.32b	50.5 ± 0.36b	5.30 ± 0.22	11.7 ± 0.47	18.3 ± 0.41	39.2 ± 0.33	43.1 ± 0.54	43.9 ± 0.51
Yes	13.1 ± 0.33a	23.1 ± 0.32a	53.5 ± 0.36a	5.92 ± 0.22	11.4 ± 0.47	18.9 ± 0.41	40.1 ± 0.33	43.0 ± 0.54	44.6 ± 0.51
Vermicompost application (B)									
0.0 t ha^−1^	11.8 ± 0.24	21.2 ± 0.40	50.3 ± 0.44c	5.25 ± 0.28	11.9 ± 0.57	18.75 ± 0.54	40.8 ± 0.40	43.1 ± 0.67	44.0 ± 0.61
5.0 t ha^−1^	12.4 ± 0.30	22.1 ± 0.40	52.1 ± 0.44b	5.90 ± 0.28	10.9 ± 0.57	17.6 ± 0.51	39.4 ± 0.40	43.0 ± 0.67	44.3 ± 0.61
10.0 t ha^−1^	12.5 ± 0.30	22.5 ± 0.40	53.5 ± 0.44a	5.75 ± 0.28	11.9 ± 0.57	19.5 ± 0.51	38.7 ± 0.40	43.1 ± 0.67	44.6 ± 0.61
F (A)	∗	∗∗	∗∗	ns	ns	ns	ns	ns	ns
F (B)	ns	ns	∗∗	ns	ns	ns	ns	ns	ns
F (A × B)	∗∗	∗∗	∗∗	ns	∗∗	∗∗	∗∗	∗∗	ns

*Note:* ± indicates the standard deviation. Values sharing the same letter within a column do not differ significantly (ns), while ∗ and ∗∗ denote significant differences at the 5% and 1% levels, respectively.

**Table 4:** Groundnut yield traits and productivity.

**Factor**	**Biomass (g plant ** ^**−1**^ **)**	**Yield components**			**Fresh yield (t ha** ^**−1**^ **)**
		**Filled pod number (pods plant ** ^**−1**^ **)**	**Filled pod weight (g plant ** ^**-1**^ **)**	**Weight of 1000 seeds (g)**	
*B. aryabhattai* M2C (A)					
No	17.5 ± 0.81b	141 ± 28.9b	259 ± 22.2b	310 ± 15.2b	9.35 ± 0.24b
Yes	18.7 ± 0.82a	176 ± 13.5a	296 ± 24.1a	338 ± 17.9a	10.1 ± 0.80a
Vermicompost application (B)					
0.0 t ha^−1^	17.1 ± 0.77b	130 ± 30.1b	247 ± 19.3b	302 ± 14.2b	9.21 ± 0.19b
5.0 t ha^−1^	18.5 ± 0.68a	172 ± 12.7a	292 ± 21.5a	333 ± 15.3a	9.81 ± 0.56a
10.0 t ha^−1^	18.7 ± 0.84a	173 ± 13.3a	294 ± 20.8a	336 ± 17.4a	10.1 ± 0.85a
F (A)	∗∗	∗∗	∗∗	∗∗	∗∗
F (AB)	∗∗	∗∗	∗∗	∗∗	∗∗
F (A × B)	ns	∗∗	ns	ns	ns

*Note:* ± indicates the standard deviation. Values sharing the same letter within a column do not differ significantly (ns), while ∗∗ denote significant differences at the 1% level.

**Table 5:** Groundnut nutrient compositions at harvest period.

**Factor**	**Seed nutrient compositions (%)**					
	**Humility**	**Lipid**	**Protein**	**Total N**	**Total P**	**Total K**
*B. aryabhattai* M2C (A)						
No	29.3 ± 1.30b	25.2 ± 0.9b	17.3 ± 0.65b	2.73 ± 0.11b	0.29 ± 0.01b	0.37 ± 0.03b
Yes	31.6 ± 2.03a	26.8 ± 1.3a	18.0 ± 0.77a	2.89 ± 0.12a	0.30 ± 0.01a	0.42 ± 0.04a
Vermicompost application (B)						
0.0 t ha^−1^	28.4 ± 0.79c	24.7 ± 0.8b	16.9 ± 0.64b	2.69 ± 0.13b	0.29 ± 0.01b	0.351 ± 0.03b
5.0 t ha^−1^	31.1 ± 1.91b	26.5 ± 1.2a	17.9 ± 0.616a	2.86 ± 0.12a	0.30 ± 0.01a	0.41 ± 0.03a
10.0 t ha^−1^	31.9 ± 1.39a	26.9 ± 1.1a	18.2 ± 0.51a	2.88 ± 0.09a	0.30 ± 0.01a	0.42 ± 0.04a
F (B)	∗∗	∗∗	∗∗	∗∗	∗∗	∗∗
F (A)	∗∗	∗∗	∗∗	∗∗	∗∗	∗∗
F (A × B)	∗∗	ns	ns	ns	ns	ns

*Note:* ± indicates the standard deviation. Values sharing the same letter within a column do not differ significantly (ns), while ∗∗ denote significant differences at the 1% level.

## Data Availability

Data availability is provided if requested.
